# Osthole Suppresses the Migratory Ability of Human Glioblastoma Multiforme Cells via Inhibition of Focal Adhesion Kinase-Mediated Matrix Metalloproteinase-13 Expression

**DOI:** 10.3390/ijms15033889

**Published:** 2014-03-04

**Authors:** Cheng-Fang Tsai, Wei-Lan Yeh, Jia-Hong Chen, Chingju Lin, Shiang-Suo Huang, Dah-Yuu Lu

**Affiliations:** 1 Department of Biotechnology, Asia University, Taichung 41354, Taiwan; E-Mail: tsaicf@asia.edu.tw; 2 Department of Cell and Tissue Engineering, Changhua Christian Hospital, Changhua 50006, Taiwan; E-Mail: ibizayeh0816@hotmail.com; 3 Department of General Surgery, Taichung Tzu Chi Hospital, Buddhist Tzu Chi Medical Foundation, Taichung 42743, Taiwan; E-Mail: guns5150@ms27.hinet.net; 4 Department of Physiology, School of Medicine, China Medical University, Taichung 40402, Taiwan; E-Mail: clin33@mail.cmu.edu.tw; 5 Department of Pharmacology and Institute of Medicine, College of Medicine, Chung Shan Medical University, Taichung 40201, Taiwan; E-Mail: sshuang@csmu.edu.tw; 6 Graduate Institute of Neural and Cognitive Sciences, China Medical University, Taichung 40402, Taiwan

**Keywords:** osthole, GBM, FAK, MMP-13, cell migration

## Abstract

Glioblastoma multiforme (GBM) is the most common type of primary and malignant tumor occurring in the adult central nervous system. GBM often invades surrounding regions of the brain during its early stages, making successful treatment difficult. Osthole, an active constituent isolated from the dried *C. monnieri* fruit, has been shown to suppress tumor migration and invasion. However, the effects of osthole in human GBM are largely unknown. Focal adhesion kinase (FAK) is important for the metastasis of cancer cells. Results from this study show that osthole can not only induce cell death but also inhibit phosphorylation of FAK in human GBM cells. Results from this study show that incubating GBM cells with osthole reduces matrix metalloproteinase (MMP)-13 expression and cell motility, as assessed by cell transwell and wound healing assays. This study also provides evidence supporting the potential of osthole in reducing FAK activation, MMP-13 expression, and cell motility in human GBM cells.

## 1. Introduction

Tumor invasion and metastasis are the main pathological characteristics of cancer cells, and mortality from cancer is primarily the result of metastatic spread to distant organs. Tumor metastasis is a highly complex multistep process that includes changes in cell-cell adhesion properties [1]. Glioblastomas (GBMs) are one of the most common and lethal types of primary central nervous system tumors, as their biological features make successful treatment very difficult. Moreover, GBM generally proves refractory to treatment by surgery, irradiation, and conventional chemotherapy. Abnormal GBM biology leads to uncontrolled growth, invasion, and angiogenesis, which ultimately facilitates cell proliferation and negatively affects survival [2–6]. Dysregulated pathways provide a design basis for molecular-targeted therapies for the treatment of gliomas, but the characteristic biology of GBM poses significant problems. Among these hurdles is the aggressive local invasion of malignant cells from the original tumor into surrounding normal brain tissue, which makes complete surgical resection impossible. Chemotherapy and ionizing radiation, alone or in combination, have produced only a modest increase in median survival. This is due to problems associated with the effective targeting of invading cells and their innate resistance to conventional therapies [5,6]. Despite advances in common treatment modalities such as surgery, radiation, and chemotherapy [7], survival in patients with these tumors has not improved [8]. Effective treatment will ultimately require a more thorough understanding of the signaling pathways that drive glioma invasion, in addition to the identification and specific targeting of critical signaling effectors.

Invasion of glioma cells into adjacent brain structures occurs through the activation of a variety of molecules including the matrix metalloproteinases (MMPs), which play an important role in tumor invasion [9]. MMPs can degrade extracellular matrix components, enabling tumor cells to invade the surrounding stroma [10,11]. MMPs can be divided into subgroups of collagenases, gelatinases, and other MMPs according to their function and substrate specificity [12]. Among the MMPs, MMP-13 is up-regulated by oncogenic proteins and activated in various cancers involving the lung, breast, prostate gland, and colon [13–15]. We previously reported that MMP-13 expression is involved in cell migration and invasion astrocytes [16], oral squamous cell carcinoma, [17] and glioma [18,19]. Importantly, clinical studies have also revealed that over-expression of MMP-13 is associated with tumor progression in glioma [20], and the highly invasive potential of cancer stem cells depends on MMP-13 enzymatic activity [21]. Recently, we have also shown that many naturally occurring food constituents and phytochemicals have diverse pharmacological efficacy, providing protective effects against a variety of cancers [22–24].

Frequent consumption of natural fruits and vegetables has been considered with reducing risk of developing various of cancer and mortality [25,26]. Osthole (7-methoxy-8-(3-methyl-2-butenyl) coumarin), an active constituent isolated from *Cnidium monnieri* (L.) *Cusson*, is represented in many Mediterranean and Middle-east regions plants and has been shown to exert a wide array of biological effects, such as contractility-based motility of different cells and tissues [27]. Osthole has also been shown to have anti-inflammatory [28], anti-osteoporosis [29], and anti-seizure [30] effects. In recent years, accumulating evidence also suggests that osthole has antitumor activities that are thought to occur via the induction of apoptosis and inhibition of cancer cell growth and metastasis [31–33]. However, effects of osthole on the migration of human GBM cells remain unclear.

This study investigates the effects and possible underlying mechanisms of osthole activity on cell death and migration in both human GBM cells and in a glioma cell line selected for a high migration potential. Our study confirms previous reports that osthole induces cell death in human glioma cells, and indicates that osthole effectively inhibits FAK activation, MMP-13 expression, and cell migration.

## 2. Results

### 2.1. Osthole Inhibits the Proliferation of Human Glioma Cells

**Figure 1.**
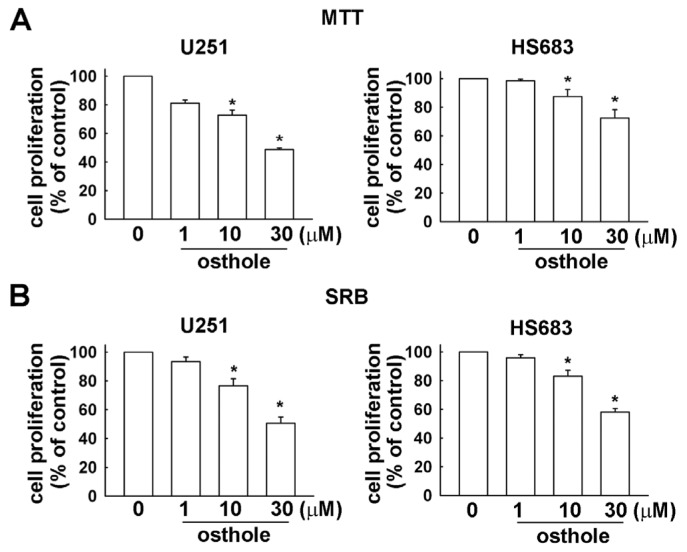
Inhibitory effects of osthole on the proliferation of human glioma cells. Cell proliferation of two different human glioma cells (U251 and HS683) is shown. Cells were incubated with various concentrations of osthole (1, 10, or 30 μM) or vehicle for 24 h and the rate of inhibition was determined by (**A**) MTT assay and (**B**) SRB assay. Results are expressed as the means ± S.E.M. of at least three independent experiments. * *p* < 0.05 compared with the vehicle treatment group.

In order to investigate the growth inhibitory effects of osthole, U251 and HS683 human glioma cells were incubated with various concentrations of osthole (1, 10 or 30 μM) for 24 h, and the rate of growth inhibition was determined by MTT and SRB assay. We observed that osthole suppressed the growth of human glioma cells in a dose-dependent manner (Figure 1).

### 2.2. Osthole Inhibits Migration of Human Glioma Cells

**Figure 2.**
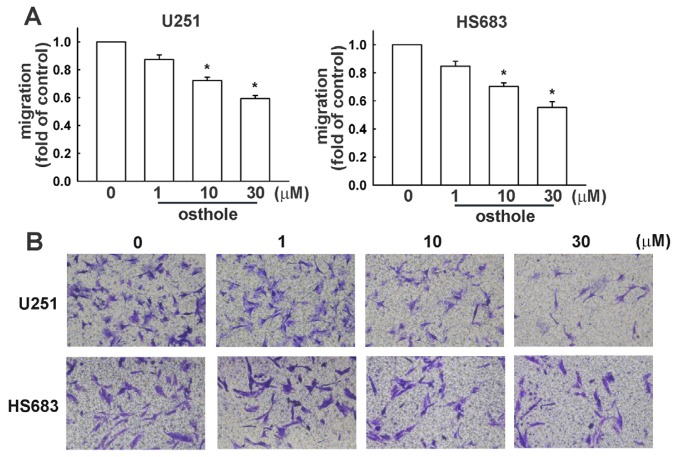
Osthole inhibits migration activity of human glioma cells. By using a cell culture insert system, *in vitro* migration activities were examined. (**A**) After incubating cells with various concentrations of osthole (1, 10, or 30 μM) or vehicle for 24 h, we found that osthole inhibited migration activity in U251 and HS683 cells. Results are expressed as means ± S.E.M. of at least three independent experiments; (**B**) Cells were treated with various concentrations of osthole or vehicle for 24 h, and migrating cells were visualized by phase-contrast imaging. Results are expressed as means ± S.E.M. of at least three independent experiments. * *p* < 0.05 compared with control group.

**Figure 3.**
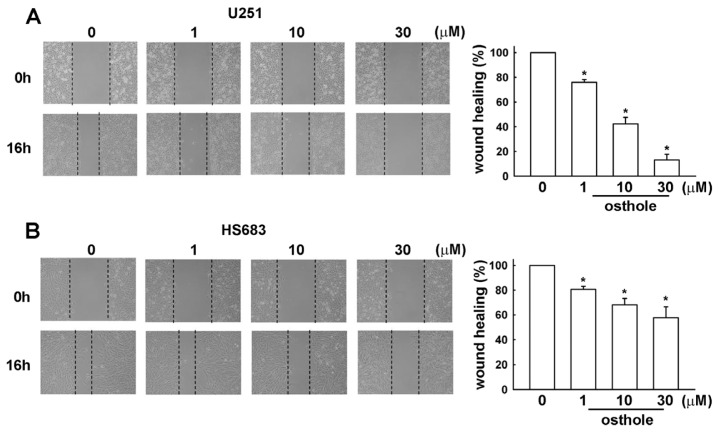
Osthole inhibits human glioma cells motility. Cells were seeded on the migration insert for 24 h and treated with various concentrations of osthole (1, 10, or 30 μM) or vehicle for another 16 h. Migrating cells were identified by wound-healing assay and visualized by phase-contrast imaging. We found that osthole inhibited cells motility in (**A**) U251 and (**B**) HS683 cells. Results are expressed as means ± S.E.M. of at least three independent experiments. * *p* < 0.05 compared with control group.

Transwell assays were performed to investigate the effects of osthole on human glioma cell migration. Osthole-regulated glioma cell migration was examined using the transwell assay. As shown in Figure 2, human glioma cells (U251 and HS683 cells, respectively) migrated from the upper to the lower chamber, and images of migrating cells are shown in Figure 2B. Our results indicate that osthole significantly inhibits human glioma cell migration in a dose-dependent manner. As shown in Figure 3, osthole also inhibits wound-healing activity in human glioma cells.

### 2.3. Osthole-Induced Inhibition of Human Glioma Cell Migration Involves MMP-13 and FAK Expression

**Figure 4.**
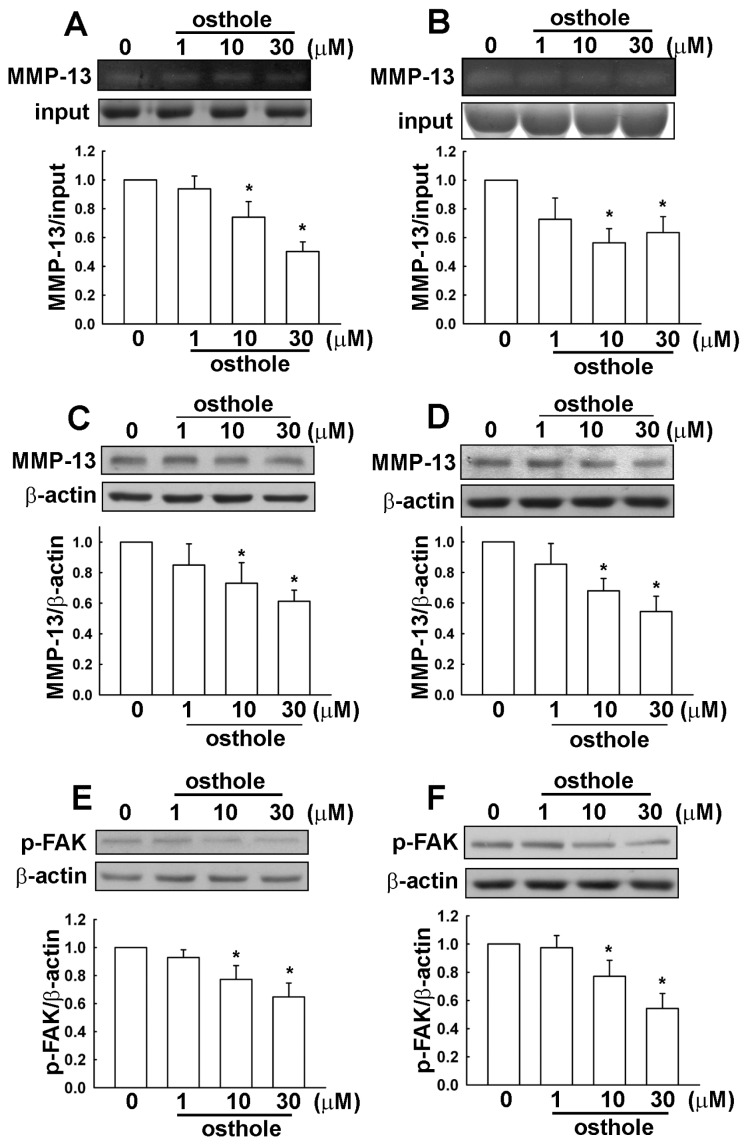
Osthole-directed migration activity involves down-regulation of MMP-13 and cell motility-dependent FAK in human glioma cells. Cells were incubated with various concentrations of osthole (1, 10, or 30 μM) or vehicle for 24 h, after which the supernatant and cell lysate extracts were collected from U251 (**A**) and HS683 (**B**) cells. MMP-13 enzymatic activities were determined by gelatin zymography (**A** and **B**); MMP-13 protein levels were determined by western blot (**C** and **D**); and phosphorylated FAK was determined by western blot analysis (**E** and **F**). Results are expressed as means ± S.E.M. of at least three independent experiments. * *p* < 0.05 compared with control group.

It has been reported that MMP-13 and FAK expression is involved in cancer cell migration. As shown in Figure 4, U251 and HS683 human glioma cells were incubated with various concentrations of osthole (1, 10, or 30 μM) for 24 h, then supernatant and cell lysate extracts were collected. MMP-13 enzymatic activities (Figure 4A,B) and MMP-13 protein levels (Figure 4C,D) were reduced after osthole administration. Moreover, phosphorylated FAK was also inhibited by osthole treatment (Figure 4E,F). The inhibition of migration activity by osthole likely involves down-regulation of MMP-13 and cell motility-dependent FAK in human glioma cells.

### 2.4. Down-Regulation of Osthole in Migration-Prone Cells

**Figure 5.**
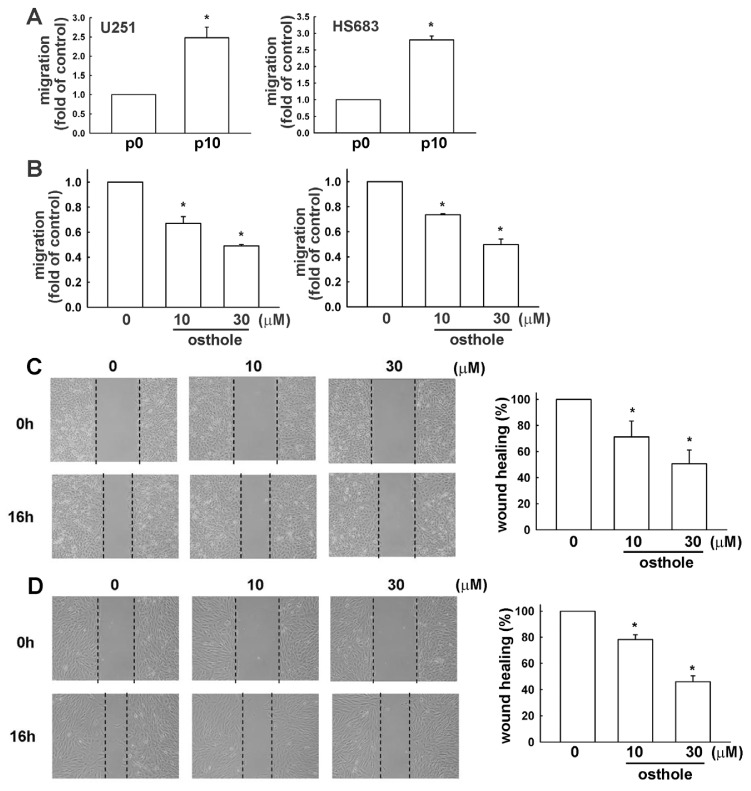
Down-regulation of osthole in migration-prone human glioma cells. (**A**) After 10 rounds of selection of U251 and HS683 cells using a cell culture insert system, the migration-prone subline (P10) exhibited higher migration ability than the original U251 and HS683 cells. Results are expressed as means ± S.E.M. of three independent experiments. * *p* < 0.05 compared with the original group (P0); (**B**) After incubating the migration-prone subline (P10) of U251 and HS683 cells with various concentrations of osthole (10 or 30 μM) or vehicle for 24 h, we found that osthole inhibited migration-prone subline (P10) migration activity in U251 and HS683 cells. Results are expressed as means ± S.E.M. of at least three independent experiments; (**C** and **D**). The migration-prone subline (P10) were seeded for 24 h and treated with various concentrations of osthole (10 or 30 μM) or vehicle for another 16 h. The cells migration were determined by wound-healing assay and visualized by phase-contrast imaging. Results are expressed as means ± S.E.M. of at least three independent experiments. * *p* < 0.05 compared with control group.

We selected U251 and HS683 cell with high cell mobility, as described in Materials and Methods. This migration-prone subline (P10) had higher cell mobility and migrated more easily through the cell culture insert basement membrane matrix than the original U251 and HS683 cells (designated as P0; Figure 5A). After incubating the P10 migration-prone subline with various concentrations of osthole (10 or 30 μM) for 24 h, we found that osthole inhibited migration (Figure 5B) and wound-healing activity (Figure 5C,D) in the P10 subline.

### 2.5. The Osthole Effects on Migration-Prone Human Glioma Cells Involve a Modulation of MMP-13 and FAK Expression

**Figure 6.**
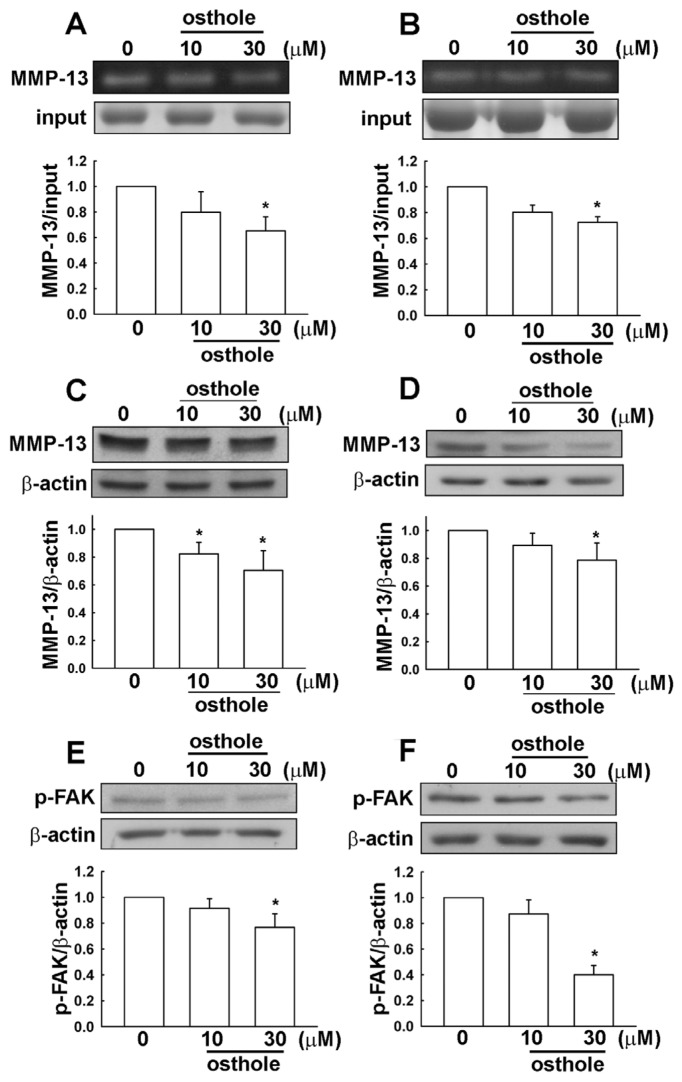
Osthole-directed migration activity involves down-regulation of MMP-13 and cell motility dependent FAK in migration-prone human glioma cells. The migration-prone subline (P10) was incubated with various concentrations of osthole (10 or 30 μM) or vehicle for 24 h, after which the supernatant and cell lysate extracts were collected in P10 cells from U251 and HS683. MMP-13 enzymatic activities were determined by gelatin zymography (**A** and **B**); MMP-13 protein levels were determined by western blot (**C** and **D**); and phosphorylated FAK was determined by western blot analysis (**E** and **F**). Results are expressed as means ± S.E.M. of at least three independent experiments. * *p* < 0.05 compared with control group.

As shown in Figure 6, The P10 migration-prone subline was incubated with various concentrations of osthole (10 or 30 μM) for 24 h, and supernatant and cell lysate extracts were then collected. MMP-13 enzymatic activities (Figure 6A,B) and protein levels (Figure 6C,D) were reduced by osthole treatment. Furthermore, the phosphorylated FAK was also inhibited after osthole administration (Figure 6E,F). We observed the down-regulation of MMP-13 and cell motility dependent FAK in P10 migration-prone human glioma cells treated with osthole.

## 3. Discussion

Glioma is the most common and aggressive type of primary brain tumor in adults, and is associated with a high mortality rate because the tumors are highly invasive and can infiltrate surrounding brain tissue, making complete surgical resection impossible [34]. In spite of enormous improvements in surgery, radiotherapy, and chemotherapy, the prognosis of glioma patients remains poor [35]. Development of experimental agents targeting glioma cells may elucidate the underlying molecular mechanisms involved in progression of the disease, and also help identify effective targets for human glioma therapies. In this study, we investigated the molecular mechanism by which osthole inhibits human glioma cell migration. Our results showed that osthole inhibits FAK phosphorylation and MMP-13 expression in human glioma cells. Importantly, osthole also inhibits FAK phosphorylation and MMP-13 expression in migration-prone glioma cells. Our previous study showed that migration-prone subline glioma cells disseminating into normal brain tissue grew orthotropically with a diffuse tumor boundary and finger-like protrusions [36]. This suggests that migration-prone subline glioma cells have enhanced migratory activity compared to normal glioma cells. Observations from the current study suggest that osthole effectively inhibits cell migration in human glioma cells, even those selected for high migratory ability.

Recent investigations suggest that osthole is a promising compound for treating a variety of central nervous system disorders, and that it can effectively cross the blood-brain barrier. Oral administration of osthole attenuates the inflammatory response in focal ischemic stroke [37], and intraperitoneal injection of osthole has a neuroprotective effect in traumatic brain injury through its antioxidative and antiapoptotic functions [38]. Administration of osthole also improves neurobehavioral function and reduces infarct volume in transient focal cerebral ischemia [39]. Furthermore, intraperitoneal administration of osthole has also been reported to protect against acute ischemic stroke due to middle cerebral ischemia occlusion [40].

MMP-13 is involved in the initiation and progression of multiple biological events required for cancer cell progression such as metastasis, invasion, and proteolytic digestion, due to its ability to digest and degrade components of the extracellular matrix [41,42]. MMP-13 also plays an important role in tumor angiogenesis and is involved in other signaling cascades leading to cancer progression [43]. A recent clinical study reported increased MMP-13 expression in glioma specimens compared with that of normal brain tissues [20]. Moreover, high expression of MMP-13 was detected more often in advanced grades of glioma [20]. Previous studies and clinical reports therefore suggest that MMP-13 can be used as a biomarker for glioma progression. Results from this study reveal that osthole inhibits MMP-13 expression in normal human glioma cells, in addition to those selected for migration-prone characteristics.

FAK is a major mechanosensitive kinase that can rapidly be activated by a variety of mechanical stimuli and plays a key role in the regulation of cell adhesion and migration [44,45]. It has also been reported that FAK functions as an intracellular tyrosine kinase that participates in a wide-ranging spectrum of biological functions such as cell proliferation and inflammation [46]. Previous studies have shown that induction of MMP-13 expression can be regulated by FAK signaling in various cell types [47,48]. It has also been reported that FAK could potentially regulate glioma cell migration [49,50]. Importantly, elevated expression of phosphorylated FAK at Tyr397 is an important feature of human glioma, and is correlated with clinicopathological features and patient survival [51]. We previously reported that phosphorylation of FAK at Tyr397 potentiates the migratory activity of human glioma cells [52]. In the present study, osthole reduced phosphoylated FAK (Tyr397) in normal human glioma cells and also in those selected to be migration-prone.

## 4. Experimental Section

### 4.1. Materials

Osthole was obtained from Sigma-Aldrich (St. Louis, MO, USA) and dissolved in DMSO (dimethyl sulfoxide). The final concentration of DMSO in this study did not exceed 0.1% and had no effect on cell viability. Fetal bovine serum (FBS) and Dulbecco’s modified Eagle’s medium (DMEM) were purchased from Gibco BRL (Life Technologies Inc., Carlsbad, CA, USA). Primary antibodies against phosphorylated FAK (Tyr397) were purchased from Cell Signaling and Neuroscience (Danvers, MA, USA). Primary antibodies specific for MMP-13 or β-actin were purchased from Santa Cruz Biotechnology (Santa Cruz, CA, USA).

### 4.2. Cell Culture

U251 and HS683 cells originated from a human brain glioma. All cell lines were purchased from the American Type Culture Collection (Manassas, VA, USA), and maintained in 75 cm^2^ flasks with DMEM. Both cell lines were cultured in medium supplemented with 10% FBS, 100 U/mL penicillin, and 100 mg/mL streptomycin at 37 °C, incubated in a humidified atmosphere consisting of 5% CO_2_ and 95% air.

### 4.3. MTT Assay

Cell viability was determined using the 3-(4,5-dimethylthiazol-2-yl)-2,5-diphenyltetrazolium bromide (MTT) assay according to our previous study [53]. U251 and HS683 cells were seeded into 96-well plates at a density of 1 × 10^4^ per well for 24 h. Following treatment with osthole for another 24 h, medium was removed and cells were washed with PBS. MTT (0.5 mg/mL) was then added to each well and the mixture was incubated for 2 h at 37 °C. MTT reagent was then replaced with DMSO (100 μL per well) to dissolve formazan crystals. After the mixture was shaken at room temperature for 10 min, absorbance was determined at 550 nm using a microplate reader (Bio-Tek, Winooski, VT, USA).

### 4.4. Sulforhodamine B Assay (SRB)

The SRB assay is based on the measurement of cellular protein content according to our previous study [54]. U251 and HS683 cells were seeded into 96-well plates at a density of 1 × 10^4^ per well for 24 h. After treatment with osthole for another 24 h, cells were fixed with 10% trichloroacetic acid and stained with SRB at 0.4% (*w*/*v*) in 1% acetic acid for 30 min. Unbound SRB was washed out with 1% acetic acid and SRB-bound cells were solubilized with 10 mM Trizma base. The absorbance was read at a wavelength of 515 nm using a microplate reader (Bio-Tek, Winooski, VT, USA).

### 4.5. Western Blot Analysis

Whole cell lysis was performed as described in our previous report [55]. Briefly, cells were treated with osthole at various concentrations then lysed with a radioimmunoprecipitation assay buffer. Protein samples were separated by sodium dodecyl sulphate-polyacrylamide gels, transferred to polyvinyldifluoride membranes, and subsequently incubated with primary and secondary antibodies [22]. The blots were then incubated in stripping buffer and re-probed for β-actin as a loading control. Quantitative data were obtained using a computing densitometer and ImageQuant software (Molecular Dynamics, Sunnyvale, CA, USA).

### 4.6. Migration Assay

The *in vitro* migration assay was performed using Costar transwell inserts (pore size, 8 mm) in 24-well plates as described previously [56]. Approximately 1 × 10^4^ cells in 200 mL serum-free media were placed in the upper chamber, and 500 mL of the same mixture containing osthole at various concentrations were placed in the lower chamber. Plates were incubated for 24 h at 37 °C in 5% CO_2_, and then cells were stained with 0.05% crystal violet and 2% methanol in PBS for 15 min. Non-migratory cells on the upper surface of the filters were removed by wiping with a cotton swab and then washed with PBS. The cell number was counted (5 fields per well) using a microscope at 100× magnification and expressed as the “fold of control”. Images of migratory cells were observed and acquired at 24 h with a digital camera and light microscope.

### 4.7. Wound-Healing Assay

Procedure of wound-healing assay was performed as described in our previous report [36]. Briefly, cells were treated with various concentrations of osthole for 16 h. A cell-free gap of 500 mm was created after removing the Ibidi Culture-Insert. Cells that migrated into the wound area were observed at 0 and 16 h with a digital camera and light microscope.

### 4.8. Gelatin Zymography

Samples of supernatant medium conditioned by cell culture under different experimental condition were centrifuged. Samples in equal volume were separated on a 10% SDS-polyacrylamide gel containing 0.1% gelatin. After electrophoresis, gels were washed with 2.5% Triton X-100 (in 50 mM Tris–HCl) for 30 min to remove SDS. Substrate digestion was formed by incubating the gel in developing buffer (50 mM Tris–Hcl containing 5 mM CaCl_2_, 1 mM ZnCl_2_, 0.02% NaN_3_, and 1% Triton X-100) at 37 °C for 24 h. Gels were subsequently stained with Coomassie brilliant blue and destained in buffer containing 50% methanol and 10% acetic acid (*v*/*v*). The location of gelatinolytic activity was detected as clear bands.

### 4.9. Establishment of Migration-Prone Sublines

Subpopulations from glioma cells were selected according to their differential migration ability; the cell culture insert system was used as described earlier. After 24 h of migration, cells that penetrated through pores and migrated to the underside of the filters were trypsinized and harvested for a second-round selection. Original cells that did not pass through membrane pores were designated as P0. After 10 rounds of selection, the migration-prone subline was designated as P10.

### 4.10. Statistics

The values given are means ± S.E.M. The significance of difference between the experimental group and control group was assessed by the Student’s *t-*test. The difference was significant if *p* < 0.05.

## 5. Conclusions

We have shown that osthole effectively induces cell death in human glioma cells, and also that this agent significantly reduces FAK phosphorylation and MMP-13 expression in human glioma cells. Cell shape is involved with cell activity and may be considered a critical determinant of cell function [57]. Recent reports also suggest that configuration of cancer cell plays a fundamental, permissive role in modulating gene expression and many important biological functions [57]. These results indicate that cell configuration may be a mediator for oncogene function during carcinogenesis and the progression of glioma. This work further demonstrates that the potency of osthole’s anticancer effect can also be observed in human glioma cells that are highly invasive. Taken together, these findings suggest that osthole could have considerable therapeutic potential in patients with glioma.
